# Low-Grade Dysplastic Nodules Revealed as the Tipping Point during Multistep Hepatocarcinogenesis by Dynamic Network Biomarkers

**DOI:** 10.3390/genes8100268

**Published:** 2017-10-13

**Authors:** Lina Lu, Zhonglin Jiang, Yulin Dai, Luonan Chen

**Affiliations:** 1 Key Laboratory of Systems Biology, CAS Center for Excellence in Molecular Cell Science, Innovation Center for Cell signaling Network, Institute of Biochemistry and Cell Biology, Shanghai Institutes for Biological Sciences, Chinese Academy of Sciences, Shanghai 200031, China; lulina@sibs.ac.cn (L.L.); jiangzhonglin@sibs.ac.cn (Z.J.); 2 Center for Precision Health, School of Biomedical Informatics, The University of Texas Health Science Center at Houston, 7000 Fannin St., Suite 820, Houston, TX 77030, USA; daiyulin@sibs.ac.cn; 3 School of Life Science and Technology, Shanghai Tech University, Shanghai 201210, China

**Keywords:** dynamic network biomarkers, hepatocellular carcinoma, low-grade dysplastic nodules, tipping point

## Abstract

Hepatocellular carcinoma (HCC) is a complex disease with a multi-step carcinogenic process from preneoplastic lesions, including cirrhosis, low-grade dysplastic nodules (LGDNs), and high-grade dysplastic nodules (HGDNs) to HCC. There is only an elemental understanding of its molecular pathogenesis, for which a key problem is to identify when and how the critical transition happens during the HCC initiation period at a molecular level. In this work, for the first time, we revealed that LGDNs is the tipping point (i.e., pre-HCC state rather than HCC state) of hepatocarcinogenesis based on a series of gene expression profiles by a new mathematical model termed dynamic network biomarkers (DNB)—a group of dominant genes or molecules for the transition. Different from the conventional biomarkers based on the differential expressions of the observed genes (or molecules) for diagnosing a disease state, the DNB model exploits collective fluctuations and correlations of the observed genes, thereby predicting the imminent disease state or diagnosing the critical state. Our results show that DNB composed of 59 genes signals the tipping point of HCC (i.e., LGDNs). On the other hand, there are a large number of differentially expressed genes between cirrhosis and HGDNs, which highlighted the stark differences or drastic changes before and after the tipping point or LGDNs, implying the 59 DNB members serving as the early-warning signals of the upcoming drastic deterioration for HCC. We further identified the biological pathways responsible for this transition, such as the type I interferon signaling pathway, Janus kinase–signal transducers and activators of transcription (JAK–STAT) signaling pathway, transforming growth factor (TGF)-β signaling pathway, retinoic acid-inducible gene I (RIG-I)-like receptor signaling pathway, cell adhesion molecules, and cell cycle. In particular, pathways related to immune system reactions and cell adhesion were downregulated, and pathways related to cell growth and death were upregulated. Furthermore, DNB was validated as an effective predictor of prognosis for HCV-induced HCC patients by survival analysis on independent data, suggesting a potential clinical application of DNB. This work provides biological insights into the dynamic regulations of the critical transitions during multistep hepatocarcinogenesis.

## 1. Introduction

Hepatocellular carcinoma (HCC) is the sixth most common cancer worldwide and the third leading cause of cancer-related deaths around the world [1]. Hepatocellular carcinoma is clinically characterized by a high incidence rate and very poor prognosis [2]. Currently, it is generally accepted that persistent hepatitis B virus (HBV) or hepatitis C virus (HCV) infections is the primary cause of chronic liver disease leading to HCC [3]. Hepatitis C virus infection is the main risk factor in western countries and Japan. Despite the progress made in numerous treatments, the survival rate of HCC patients remains low because HCC is not easily detected prior to the advanced stage. Thus, it is of utmost importance to clinically diagnose early HCC.

**Figure 1:**
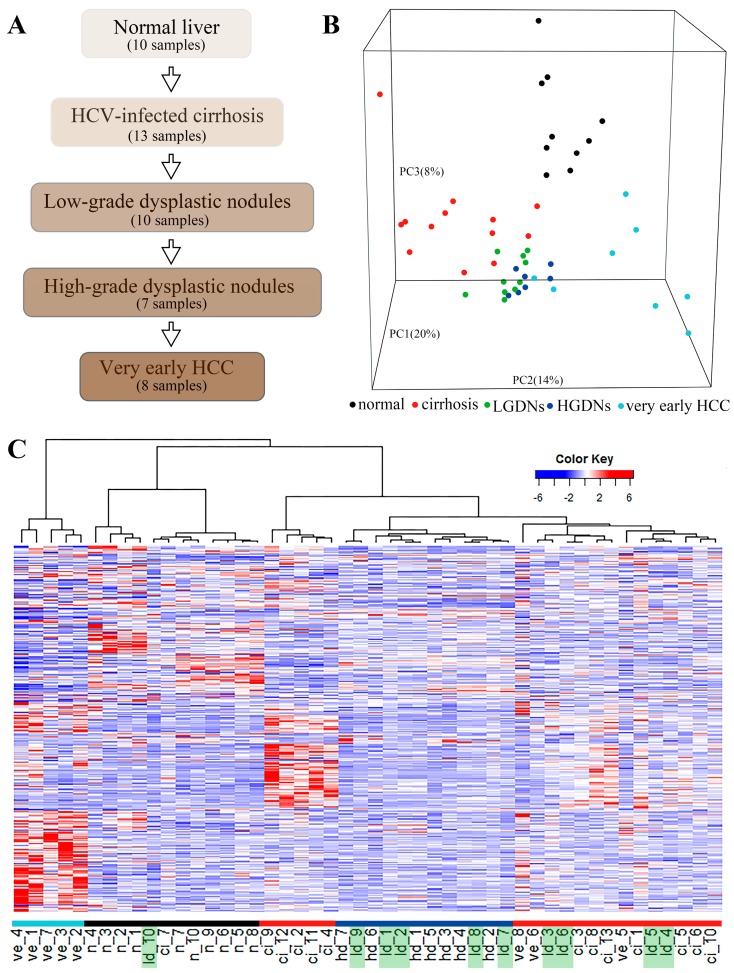
The progression of hepatitis C virus (HCV)-induced hepatocellular carcinoma (HCC) and gene expression profiling. (**A**) A schematic diagram shows the HCV-induced HCC development. (**B**) A three-dimentional image shows principal component analysis (PCA) for clustering 48 samples along HCC development. Each small spot represents the principal component (PC) score along the top three principle components for each sample. (**C**) Unsupervised hierarchical clustering of 48 tissue samples using the Pearson correlation coefficient (PCC) distance. Similar to (**B**), low-grade dysplastic nodules (LGDNs) samples are not grouped together but dispersed into cirrhosis and high-grade dysplastic nodules (HGDNs) groups. n: Normal; ci: Cirrhosis; ld: Low-grade dysplastic nodules; hd: High-grade dysplastic nodules; ve: Very early HCC. The colored bars mark clusters: black, normal; red, cirrhosis; dark blue, HGDNs; light blue, Very early HCC. Dispersed LGDNs samples are highlighted in green.

In recent years, the concept of multi-step human hepatocarcinogenesis has been well documented [4,5,6]. The liver injury induced by HCV produces a progressive inflammatory milieu that results in a cycle of necrosis and regeneration leading to liver cirrhosis. Subsequently, cirrhosis patients often present dysplastic nodules. These lesions which are confirmed as precancerous lesions of HCC are classified as low-grade dysplastic nodules (LGDNs) and high-grade dysplastic nodules (HGDNs) based on presence of cytologic and architectural atypia [7]. Although the morphology of these nodules is not sufficient to support a diagnosis of malignant tumor, these nodules are closely correlated with the occurrence of HCC. And, the HGDNs are more likely transformed into HCC than LGDNs based on clinical, pathological, molecular genetics, and radiological assessments [8,9,10,11]. The sequence of HCC initiation and progression is shown in Figure 1A, but the precise molecular events and their regulatory networks that underlie HCC formation remain largely unknown.

**Figure 2:**
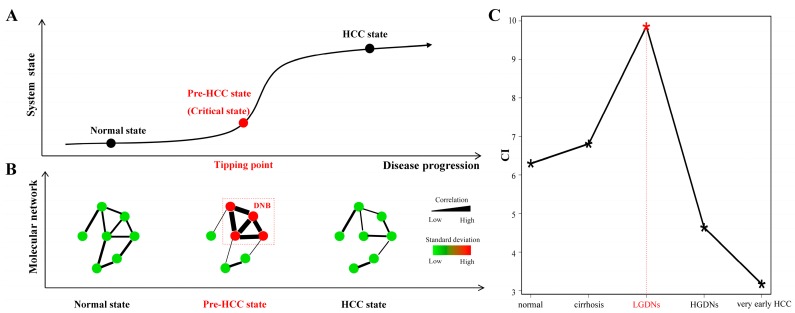
A brief mathematical model of dynamic network biomarkers (DNB) theory and its analysis results. (**A**) Three stages during HCC progression. A normal state is a relatively healthy stage in which the disease is under control, whereas the pre-HCC state or the critical state at the tipping point is the limit of the normal state just before the transition of the disease. After the tipping point, the system drastically deteriorates to the disease state. The DNB method can identify the pre-HCC state at the tipping point by using the signals shown in (**B**). (**B**) Dynamic network biomarkers as a network signals the emergence of the critical transition. When the system approaches the pre-HCC state, DNB members satisfy the three conditions. The expression of DNB members become strongly fluctuate (high standard deviations), and these DNB members are highly correlated, meanwhile, the correlations between DNB members and other non-DNB members decrease. Here, edge width corresponds to the correlation between a pair of nodes, and node color corresponds to the standard deviation of a node. (**C**) Scores of candidate DNBs in every stage. The score in the LGDNs stage is obviously higher than other stages, therefore, the molecule module in LGDNs is considered as the DNB and the LGDNs correspond to the tipping point during the HCC progression.

Recently, a novel, model-free approach based on nonlinear dynamic theory, termed dynamic network biomarkers (DNB), was developed to detect critical transitions or tipping points during the progression of complex diseases [12,13]. Generally, a disease progression can be divided into three stages, i.e., normal state, critical state (or the tipping point), and disease state (Figure 2A). After the tipping point moves gradually from the normal state, the system drastically deteriorates to a disease state. Specifically, DNB is a group of molecules (i.e., genes, RNAs, proteins, or metabolites) with strongly collective fluctuations. Based on nonlinear dynamical theory, DNB appears only at the tipping point of a homeostatic system and the molecules in DNB are strongly correlated and also fluctuated just before the critical transition (i.e., the tipping point or pre-disease state). Quantitative criteria for the DNB can be obtained by measuring the differential correlations and deviations of molecular expressions rather than the differential expressions adopted in the traditional methods. In contrast to the “disease diagnosis” by traditional biomarkers, DNB is for “disease prediction” (i.e., for the pre-disease diagnosis as the early-warning signals of the disease state). If the state of a system passes over the tipping point to the disease state, it becomes very difficult to reverse to the normal state even by advanced medical treatment. Therefore, it is crucial to identify the pre-disease state so as to prevent the irreversible deterioration of the disease. In addition to complex diseases, DNB theory had also been applied to detect the tipping points in cell fate decisions and immune checkpoint blockade processes [14,15].

Given the difficulty to diagnose early HCC, it is a key problem to identify when and how the tipping point or the critical transition happens at a molecular level. In this work, from stage-wise gene expression profiles of HCC initiation (i.e., normal, cirrhosis, LGDNs, HGDNs, and very early HCC), we identified the tipping point or pre-HCC state of HCV-induced HCC by DNB model. The obtained DNB formed a specific module with 59 genes at the LGDNs stage to signal the tipping point just before the drastic deterioration in HCC progression. We also partially revealed molecular mechanism on the HCC initiation by functional analysis of DNB, which both provides biological insights into the dynamic regulations of the critical transitions and opens a new way for the identification of therapeutic targets. We further identified biological pathways responsible for the critical transition, including several pathways in immune system reactions, cell growth and death, and cell adhesion. And furthermore, DNB was validated as an effective predictor of prognosis for HCC patients by survival analysis on independent data.

## 2. Materials and Methods

### 2.1. Gene Expression Datasets

The gene expression profiles for DNB analysis were obtained from the Gene Expression Omnibus database (GEO, https://www.ncbi.nlm.nih.gov/geo/) under accession ID GSE6764. And the normalized expression values processed by marker aided selection (MAS) method were downloaded. The dataset includes the expression profiles of 75 tissue samples (10 normal liver tissues, 13 cirrhosis liver tissues, 10 LGDNs, 7 HGDNs, 8 very early HCC, 10 early HCC, 7 advanced HCC and 10 very advanced HCC) from 48 patients with HCV infection representing the stepwise carcinogenic process from preneoplastic lesions to HCC. The earlier five stages were selected to study pathogenesis of HCC. In this dataset, probe sets without corresponding gene symbols were excluded during our analysis, while multiple probe sets mapping to the same gene were averaged as the expression values. And for the probe sets mapping to more than one gene, we took the first annotation.

As an independent dataset for validation, a cohort of HCC patients was subjected to survival analysis. The dataset was deposited in International Cancer Genome Consortium (ICGC) database (https://icgc.org/) provided by RIKEN (project code: LIRI-JP) including 260 donors [16]. Both the gene expression profiles (FPKM data) and clinical-pathological information were downloaded. The data from patients without HCV infection were not considered in this study.

### 2.2. Identification of Dynamic Network Biomarkers

It has been indicated that the progression of many chronical diseases (e.g., cancer) is not always smooth but there is an abrupt change after a system state passes over a critical state or pre-disease state, resulting in the drastic transition or serious deterioration to a disease state. However, in contrast to the significant difference between normal state and disease state in terms of molecular concentrations (or differential expressions of proteins or genes), there is generally no significant difference between normal state and pre-disease state. Hence, traditional molecular biomarkers or methods may fail to diagnose the pre-disease state. To overcome this problem, based on nonlinear dynamical theory, the DNB method was proposed to detect the pre-disease state or critical state by exploring fluctuation information of the measured omics data, rather than the information of traditional differential expressions [12]. In brief, DNB is a group of molecules satisfying the following three requirements when the system approaches the critical state:

Condition 1: The DNB members are closely correlated to each other, i.e., their average Pearson correlation coefficient (*PCC_in_*) in an absolute value becomes very high.
Condition 2: The DNB members lose correlations with other non-DNB members, i.e., the average PCC (*PCC_out_*) between DNB members and non-DNB members becomes very low.
Condition 3: The DNB members are highly fluctuated, i.e., their average standard deviation (*SD_in_*) becomes very high.

Based on nonlinear dynamical theory, whenever DNB satisfying all above three criteria appears, the system is at the tipping point. Therefore, the three conditions are considered as the generic properties to detect early-warning signals of the pre-disease state or critical state. Note that DNB is a functional module, which signals the imminent transition or deterioration from the normal state to the disease state, and, therefore, is considered to be causally related to the initiation and progression of the disease [12,13,17,18].

The three conditions can be combined into a single composite index (*CI*) to quantitatively detect the DNB as follows:

(1) $$CI=\frac{SD_{in}\times PCC_{in}}{PCC_{out}}$$

where *SD_in_* and *PCC_in_* are the average standard deviation and average absolute PCC of all molecules in DNB, corresponding to Conditions 3 and 1, respectively. The *PCC_out_* is the average absolute PCC between molecules inside and outside of DNB, corresponding to Condition 2.

Assume that we collect molecular profiles of several samples (e.g., gene expression data) in each stage *t* during the disease progression process (total T stages, i.e., *t* = 1, …, T). The detailed flow of the computational algorithm for detecting DNB as well as the tipping point as follows:

1. For each stage *t*, calculate PCCs for all pairs of genes or molecules with respect to the samples. The PCC at the 0.05 quantile of the descending order PCCs among all pairs was regarded as the threshold for a high PCC at each stage.
2. For each stage *t*, select the genes with their standard deviations above 50% percentile based on gene expression profiles. Then we get a module of molecules at each stage as M_t_.
3. Hierarchically cluster the M_t_ to get smaller modules whose molecules have high correlations at each stage. The distance used in clustering is defined as 1−|PCC|, and the cutoff is set based on the significance test for PCC in step one. Denote the resulting modules at each stage *t* as *C_t_* = {*c_t_*}.
4. For any module *c_t_*∈*C_t_*, calculate its *CI* and find the module with the maximum index at each stage as the candidate DNB.
5. Find the module with the maximum *CI* value among all T stages with T candidate DNBs. This module is our DNB and the corresponding stage is the tipping point, at which the system is considered at the pre-disease or critical state.

### 2.3. Samples Clustering

Unsupervised hierarchical clustering of all samples along development of HCC was performed by the PCC distance based on differentially expressed genes (DEGs) assessed using one-way analysis of variance (ANOVA) (*p* < 0.01) [19]. Visualization of the Samples clustering by heat map was achieved by using gplots package of R 3.2.5 (http://www.R-project.org/). Principal component analysis (PCA) was performed using the prcomp function in the stats package from R software.

### 2.4. Functional Analysis

During functional enrichment analysis of genes, the online software Database for Annotation, Visualization, and Integrated Discovery (DAVID 6.8, https://david.ncifcrf.gov/), clusterProfiler package and signaling pathway impact analysis (SPIA) package of R were utilized to perform Gene Ontology (GO) analysis and Kyoto Encyclopedia of Genes and Genomes (KEGG) pathway enrichment analysis [20,21]. Terms with *p*-values under 0.05 after correction for multiple hypotheses testing by false discovery rate (FDR) were considered as significantly enriched. Ingenuity Pathway Analysis (IPA) software 01-10 (Ingenuity Systems, Redwood City, CA, USA) was also used for pathways enrichment analysis. Those genes with known gene symbols were uploaded into the software and each gene symbol was mapped to its corresponding gene object in the Ingenuity Pathways Knowledge Base. Significance threshold of Student’s *t*-test was set at 0.05. To further analyze the functions of the DNB, STRING resource (https://string-db.org/) was utilized for protein–protein interactions (PPIs) network analysis [22] and all networks were visualized by Cytoscape software 3.5.1 (http://www.cytoscape.org/). In the network, each gene was corresponded to a node and the nodes connected by edges (lines). The degree of a gene was defined as the number of the first neighbor genes linked to it within a network, which can assess the relative significance of a gene in the network. Thus, in a network, the more the first neighbor genes a DNB member connects, the higher degree it has and the more important it is.

### 2.5. Statistical Analysis

Two-tailed, unpaired Student’s *t*-tests were used for identifying DEGs between two stages. Genes with FDR adjusted *p* < 0.05 were considered to be differentially expressed. Survival curves were generated using Kaplan–Meier estimates and differences between the curves were evaluated by log-rank test. Differences were considered as statistically significant when *p* < 0.05.

## 3. Results

### 3.1. Gene Expression Profiling

The generally chronologic sequence of HCV-induced HCC is shown in Figure 1A. Patients with HCV infection first develop chronic hepatitis leading to liver cirrhosis, subsequently to LGDNs and HGDNs, and finally to HCC. The samples with five stages including 10 normal liver tissues, 13 cirrhosis liver tissues, 10 LGDNs, 7 HGDNs, and 8 very early HCC in GSE6764 were studied in this work. We compared the gene expression profiles of these 48 tissue samples using the PCA. A three-dimensional PCA was performed, representing 42% of the data information (Figure 1B). Clearly, almost all histological-stage samples were segregated from each other except LGDNs. The LGDN samples were not clustered together and dispersed, suggesting their special stage. To further analyze the correlation between the expression profiles and pathological stages, an unsupervised hierarchical clustering based on 4340 DEGs was performed by the PCC distance and visualized via a heat map (Figure 1C). Each group is distinguished by a different color. The first branch allowed the separation of very early HCC from non-carcinoma samples. A sub-branch partitioned the non-carcinoma samples between normal samples and preneoplastic lesions. In the precancerous cluster, the samples in cirrhosis and HGDNs were clustered together respectively, while LGDNs (Figure 1C, in green) samples were not grouped together but dispersed into cirrhosis and HGDNs groups. The result of the hierarchical clustering was consistent with the three-dimensional PCA, which also implied that LGDNs was special and different from other stages.

### 3.2. Dynamic Network Biomarkers Theory Detects Low-Grade Dysplastic Nodules as the Tipping Point during Hepatocarcinogenesis

Identifying the pre-HCC state at the tipping point during HCC progression is crucial for diagnosis and treatment purpose (Figure 2A). A DNB model was developed by measuring fluctuations and correlations of a group of molecules rather than their differential expressions, and this is the first theoretical work to quantitatively detect the critical state only based on the observed data (see Materials and Methods). The three criteria for DNB members are summarized in Figure 2B, i.e., at the tipping point or pre-disease state, (i) the expressions of DNB members become highly fluctuated (high standard deviations); (ii) these DNB members are highly correlated (high PCCs) and (iii) correlations between DNB members and other non-DNB members disappear (low PCCs).

Based on the above three criteria, we conducted the DNB analysis to detect the pre-HCC state (see Materials and Methods). Then, we got five candidate DNBs at five different stages and the scores of the candidate DNBs were shown in Figure 2C. Clearly, the score of the candidate DNB at LGDNs was significantly higher than the other four-time points, so this candidate DNB was considered as the real DNB for signaling the drastic deterioration during the progression of HCC. Thus, the disease state of HCC would present after the tipping point (i.e., LGDNs stage). It was consistent with previous reports: after LGDNs stage, the disease was drastically deteriorated to HGDNs, which was considered closest to HCC based on histopathological features and clinical follow-up studies with a high risk of transformation [8,9]. The three parameters (*SD_in_*, *PCC_in_*, and *PCC_out_*) of obtained DNB were satisfied with typical features of the critical state characterized by DNB (Supplementary Figure S1). The DNB included 59 genes (Supplementary Table S1) and some of them have been reported as related to HCC initiation. For instance, tripartite motif containing 21 (TRIM21) which has E3 ligase activity and functions in the process of ubiquitination and is a potential tumor suppressor in HCC [23]. Interferon-stimulated gene 15 (ISG15) high expression is an intrinsic feature for HCC and a trigger for tumorigenesis and metastasis [24,25]. The network of DNB was constructed based on high confidence protein–protein interactions from the STRING database. As shown in Supplementary Figure S2, strong functional relationships existed among DNB members.

### 3.3. The Key Biological Processes in which Dynamic Network Biomarkers are Involved

Based on DNB theory, the DNB subnetwork as the early-warning signal was strongly related to disease development. For analyzing the mechanism of DNB at a network level during hepatocarcinogenesis, we firstly performed the GO enrichment analysis and KEGG pathway enrichment analysis on 59 DNB members (Supplementary Table S2).

Type I interferon signaling pathway, response to virus, and negative regulation of viral genome replication were the most relevant biological processes, validating the significant correlation between the disease evolution and the DNB [26,27]. Except for processes involving immune response, protein polyubiquitination was also detected for playing a role in the critical transition to HCC, which participates in many processes important for cellular homeostasis such as regulation of the cell cycle, apoptosis, endocytosis, and many more [28,29]. In addition, at the KEGG pathway enrichment level, the pathway of hepatitis C and viral carcinogenesis was among the top five, which directly indicated the close correlation between the DNB and HCV-induced HCC. Meanwhile, many other pathways related to immune system reactions, such as antigen processing and presentation, the nucleotide-binding oligomerization domain-containing protein (NOD)-like receptor signaling pathway, and the RIG-I-like receptor signaling pathway were listed in the results. The TGF-β signaling pathway and JAK–STAT signaling pathway participating in regulating cell proliferation, differentiation, cell migration, and apoptosis were also significantly enriched, which were dysfunctional processes involved in fibrogenesis and progression of HCC [30,31,32]. Consistently, many canonical pathways related to immune system reaction were enriched in the result of ingenuity pathway analysis (IPA) (Supplementary Table S3). These pathways and biological processes played remarkable roles in early carcinogenesis and partially bringing about the critical transition to HCC. Furthermore, many genes in the DNB took part in more than one pathway, such as human leukocyte antigen (HLA)-A, HLA-B, HLA-C which were identified from six enriched KEGG pathways and these genes could affect the cross-talking among different pathways.

### 3.4. Dynamic Network Biomarkers Play Key Functional Roles in Coordinating the Critical Transition

**Figure 3:**
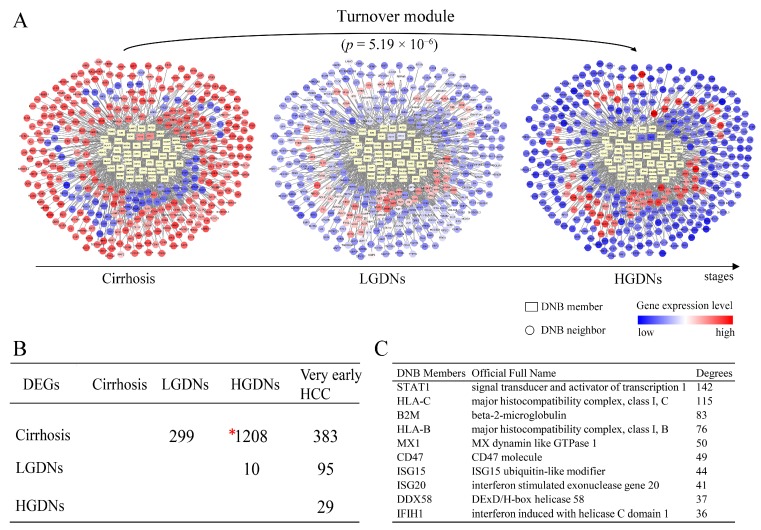
The DNB-associated network involving differentially expressed genes (DEGs) and dynamical changes of DEGs before and after the critical state. (**A**) These series of networks visually demonstrate the dynamic changes of DNB-associated network in terms of expressions before and after the critical state. There are 360 DEGs linked to DNB in the network, which significantly change from low (or high) at cirrhosis to high (or low) at HGDNs. Differentially expressed genes are significantly enriched in DNB-associated network with *p* = 5.19 × 10^−6^ by hypergeometric test. (**B**) The numbers of DEGs between any two stages during hepatocarcinogenesis. Genes with FDR adjusted *p* < 0.05 are considered to be differentially expressed by Student’s *t*-tests. One thousand two hundred eight DEGs between cirrhosis and HGDNs highlight the huge differences before and after the tipping point. (**C**) The top 10 DNB members with high degrees in the network.

For further analyzing the mechanism behind the drastic deteriorations during hepatocarcinogenesis, 1208 DEGs before and after the critical period were picked up by Student’s *t*-test statistics with *p* < 0.05 after FDR correction. The largest amount of DEGs between different disease stages (i.e., cirrhosis and HGDNs), highlighted the stark differences before and after the tipping point (Figure 3B), which implied the drastic deterioration phenomena after LGDNs. The DNB members and DEGs had been linked and correlated by the combined functional linkages from protein–protein interactions database STRING and visualized by Cytoscape (www.cytoscape.org/) (Figure 3A). Three hundred sixty DEGs which significantly changed (or inversed) from low (or high) at cirrhosis stage to high (or low) at HGDNs stage were linked to 48 DNB members directly. Although only two DNB members, ring-box 1 (RBX1) and interferon stimulated exonuclease gene 20 (ISG20) belonged to these 1208 DEGs, the DEGs were significantly enriched in DNB-associated network with *p* < 0.01 by hypergeometric test (Figure 3A), implying their functional relations, especially during the critical period.

The degree of each DNB member was defined as the number of the first neighbor genes linked to it within the network, which could assess the relative significance of the DNB member in the network. The top ten DNB members with the highest degrees are listed in Figure 3C. The STAT1 acts as a transcription factor mediating cellular responses to cytokines and growth factors, playing a pivotal role in cell cycle and cell fate determination. Activation of STAT1 induces a variety of antiviral proteins which inhibit HCV replication. The STAT1 has been reported to play a critical role in pathogenesis of liver diseases [27,33,34]. β-2-microglobulin (B2M), HLA-C, and HLA-B are components of the class I major histocompatibility complex (MHC), involved in the presentation antigens to the immune system. The HCV core protein induces MHC class I upregulation to achieve immune evasion [35]. The MX1 protein is a GTPase that acts at an early step of the virus life cycle, prior to the genome replication, by trapping viral components and preventing them from reaching their cellular destination. Also, MX1 is reported to be related with HCV clearance during acute HCV infection and after interferon therapy, and severity of liver disease [36,37]. Cluster of differentiation (CD) 47 is a cell surface molecule that inhibits phagocytosis of cells which express it by binding to its receptor on macrophages and other immune cells. The CD47 is expressed at different levels by normal and neoplastic cells. Targeting CD47 is in the spotlight of immunotherapy in lung and breast cancer [38,39]. The degrees of whole DNB members in the network are shown in Supplementary Table S4.

**Figure 4:**
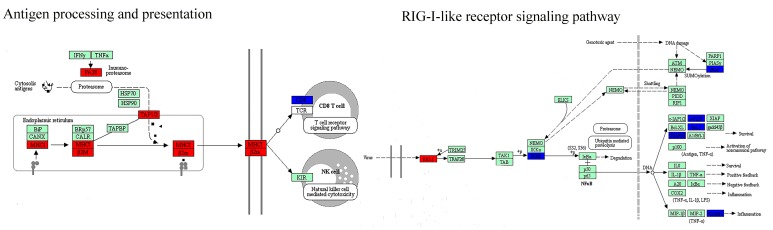

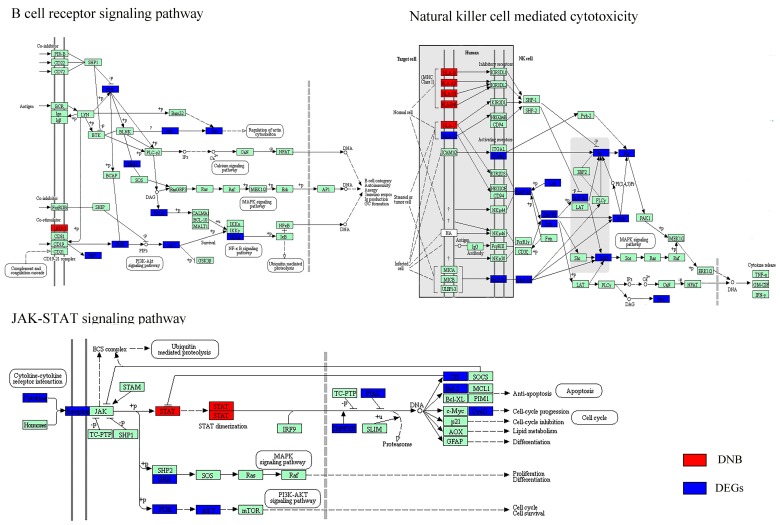
Hepatocellular carcinoma-related biological pathways with both DNB members and DEGs. DNB members are placed at relatively important positions in the pathways (e.g., transciption factors, receptors, and upstream regulators). DNB: dynamic network biomarkers; DEGs: differentially expressed genes.

At a network level, DEGs were significantly enriched in DNB-associated network (Figure 3A) which indicated the strong relationships between DNB and DEGs. Furthermore, we detected the connection at a pathway level. The net perturbation accumulation (Acc) from the SPIA method [21] was used to estimate the overall perturbation of direct upstream genes on one gene, which can be used to detect the relative position between DEGs and DNB members. We set the fold changes of DEGs and DNB members to SPIA. Among the 12 significantly enriched HCC-related pathways, we found that the Acc of DNB members was 0 (except for the JAK–STAT signaling pathway), while the Acc of DEGs varied by pathway, indicating the DNB members suffer less perturbation than DEGs do from upstream genes (Supplementary Table S5). From this result, we could infer that a DNB was located relatively upstream of these pathways. Then, we specifically mapped the DNB and DEGs to KEGG pathways. We also found that in the HCC related pathways which contained genes from both DNB and DEGs, the members of DNB stayed at relatively important places (Figure 4). For instance, several DNB genes were located upstream of RIG-I-like receptor signaling pathway and many downstream genes presented significantly altered expression. Some other DNB genes as receptors of pathways can help signal transduction (e.g., B cell receptor signaling pathway and natural killer cell-mediated cytotoxicity). And, STAT1 as a transcription factor made a significant impact on JAK–STAT pathway.

### 3.5. Biological Functions Influenced by Dynamic Network Biomarkers and Differentially Expressed Genes before and after the Critical Transition

To further understand the types of pathways that were infected during the critical transition, the SPIA method was used, which measured the actual perturbation on a given pathway under a given condition [21]. Although most of the DNB members have no significantly altered expression before and after the critical transition, the enriched pathways (*p* < 0.05) based on DNB and DEGs were similar. Four overlaps and close crosstalk between DNB and DEGs could be detected (Table 1). The cytosolic DNA-sensing pathway belonging to the enriched pathways by DNB included the nuclear factor-κB (NF-κB) signaling pathway, which was in the pathways enriched by DEGs. This relation also existed for cell adhesion molecules (CAMs) and tight junctions. Moreover, mitogen-activated protein kinases (MAPK) signaling pathway belonging to the enriched pathways by DEGs was downstream of several pathways enriched by DNB, e.g., RIG-I-like receptor signaling pathway, natural killer cell mediated cytotoxicity, the JAK–STAT signaling pathway, and the TGF-β signaling pathway.

As shown in Table 1, pathways of both sides participated in the same biological functions and their regulation direction was consistent. During the critical transition, biological function including cell growth and death, immune response, and cell adhesion were significantly regulated. Pathways involved in immune response and cell adhesion were downregulated, such as the JAK–STAT signaling pathway, NF-κB signaling pathway, and RIG-I-like receptor signaling pathway. Meanwhile, pathways related to cell growth and death, such as cell cycle, the Notch signaling pathway, and Hedgehog signaling pathway were upregulated. These biological functions could be responsible for the critical transition from preneoplastic lesions to HCC.

### 3.6. Prognostic Analyses of Dynamic Network Biomarkers

Dynamic network biomarkers played a key functional role during HCC onset and we found that 43 of the 59 DNB members were differently expressed with *p* < 0.05 between normal tissues and HCC tissues, indicating the efficacy of DNB members in HCC progression. To detect whether or not DNB has potential in clinic practice, survival analysis was performed based on 122 HCV-induced HCC patients with gene expressions and clinical information from the Liver Cancer–RIKEN, JP, Japan (LIRI-JP) project in the ICGC database.

**Figure 5:**
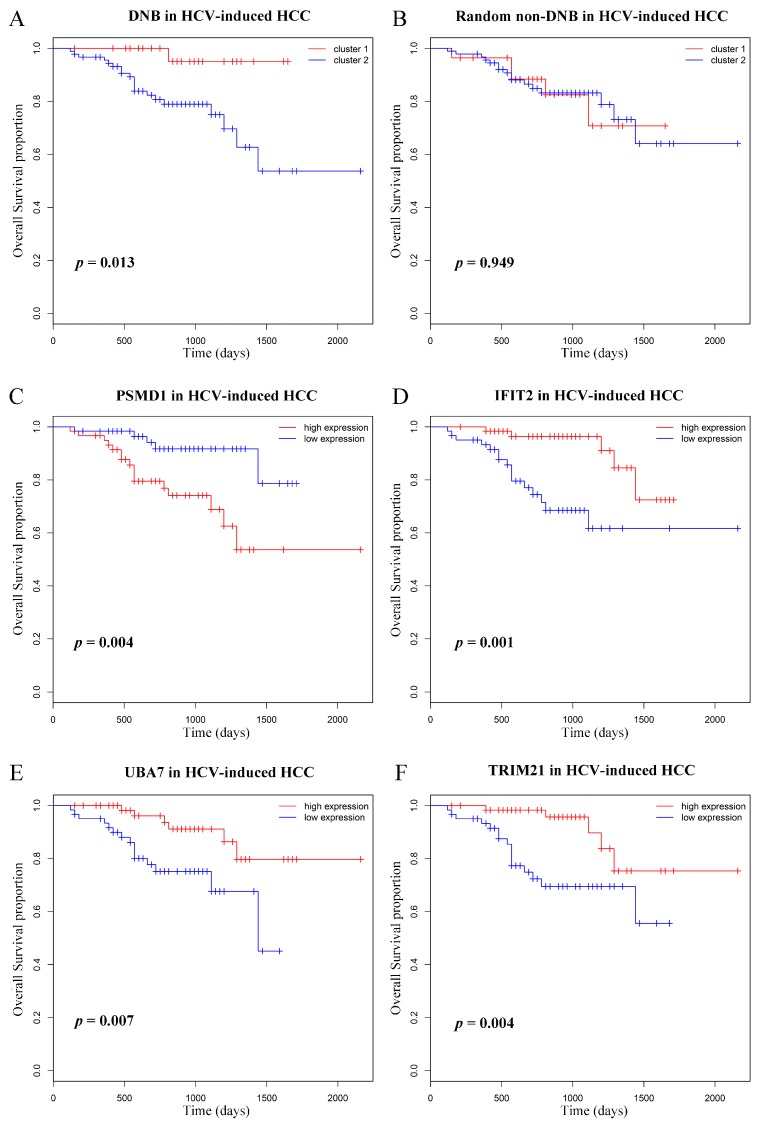
Kaplan–Meier overall survival curves for the HCV-induced HCC patients based on DNB. Illustration of prognostic difference between the two groups identified by the DNB (**A**) and by random non-DNB (**B**). (**C**–**F**) The survival curves of patients with different expression levels of representative DNB members. Patients were divided into low- and high-expression groups according to the median value.

According to the expressions of genes in DNB, the HCV-induced HCC patients were separated into two groups by k-means clustering, and group 2 displayed obviously poor survival, as shown in Figure 5A. As a comparison, expression of the randomly chosen non-DNB genes were also used to predict the prognostic difference, which failed to discover the biologically meaningful groups (Figure 5B). Subsequently, we investigated the independent prognostic significance of each member in DNB and found 14 of them could predict patient survival with *p*-values < 0.05. Figure 5C–F displays four representative genes with significant survival values, and the others are shown in Supplementary Figure S3.

## 4. Discussion

In this study, we identified a critical stage (or pre-HCC) just before the crucial transition from preneoplastic lesions to HCC based on multi-stage gene expression profiles during HCC progression by DNB theory. Unlike traditional molecular biomarkers which usually distinguish disease state from normal state, DNB can detect the critical state just before the disease state to realize early. Although the DNB method is a model-free approach, it generally requires multiple samples in each sampling period so as to detect the tipping point. Based on the three conditions of DNB, LGDNs was revealed as the tipping point during multistep hepatocarcinogenesis. It was consistent with reports in the literature that found that after the LGDNs stage, the disease drastically deteriorated to HGDNs, which is the closest manifestation to HCC, based on histopathological features and clinical follow-up studies with a high risk of transformation [5,10,40]. Although each step in HCC progression could be a target for prevention of HCC, intervention before the tipping point could be more practical.

It should be noted that the DNB subnetwork is not necessarily a set of driving factors [12], but provides the early-warning signals of the pre-HCC state, thus predicting the upcoming HCC onset before the occurrence of the disease phenotype based on its dynamic features. Moreover, DNB can also be implemented to analyze the underlying molecular mechanisms of disease initiation at a network level. The functional enrichment of GO biological processes and pathways validated the significant correlation between hepatocarcinogenesis and the DNB. Most of the enriched terms were related to inflammation, immune responses, cell proliferation, differentiation, cell migration, and apoptosis, such as negative regulation of viral genome replication, viral carcinogenesis, the type I interferon signaling pathway, RIG-I-like receptor signaling, TGF-β signaling pathway, and JAK–STAT signaling pathway. These analyses indicate that these biological processes and pathways play a remarkable role resulting in hepatocarcinogenesis, and the genes in DNB make them move to the disease phenotype on HCC initiation.

In DNB, only two genes presented significantly differential expressions between cirrhosis and HGDNs. However, 43 DNB members were differently expressed between normal tissues and HCC tissues, implying the effectiveness of DNB members in HCC progression. The effect of DNB at HCC onset depended not on their differential expressions, but on collective fluctuations according to DNB theory. Thus, we detected the relation between DNB and DEGs during critical transition at a network level. The DEGs before and after the tipping point were significantly enriched in the DNB-associated network, further implying that DNB plays core functional roles in coordinating the critical transition from preneoplastic lesions to HCC, which results in drastic deterioration phenomena after LGDNs. We also found that in the HCC related pathways which contained genes from both DNB and DEGs, several DNB members stayed at relatively important places, such as receptors, upstream regulators and transcription factors. Hence, we hypothesized that the emergence of DNB modules made the expression of those closely related genes change considerably and caused several vital biological processes to become abnormal, ultimately leading to the drastic deterioration to the irreversible HCC state. Although only two DNB members (RBX1 and ISG20) belonged to DEGs, the pathways significantly regulated by DNB and DEGs were similar. During the critical transition, pathways in immune response and cell adhesion were down-regulated, such as the JAK–STAT signaling pathway, NF-κB signaling pathway, and RIG-I-like receptor signaling pathway. Meanwhile, pathways related to cell growth and death, such as cell cycle, the Notch signaling pathway, and Hedgehog signaling pathway were up-regulated. Moreover, it had been reported that immune response and cell adhesion were upregulated in cirrhosis and downregulated on HCC initiation, and cell proliferation was upregulated on HCC initiation [41]. It could be inferred that the HGDNs stage was very close to HCC onset and demonstrated the LGDNs stage as the tipping point during hepatocarcinogenesis.

Furthermore, we performed survival analysis based on 122 HCV-induced patients for detecting whether or not DNB has clinical application value. The results show that DNB is effective as a clinical predictor of prognosis for HCV-induced HCC patients. In conclusion, DNB during hepatocarcinogenesis can be used as early-warning signals of HCC, and this work also opens a new way to understand the underlying mechanisms responsible for HCC initiation and provides a new method to facilitate the identification of molecular targets. This method can also be applied to the analysis of other diseases [42,43,44] in a similar manner.

## Figures and Tables

**Table 1:** Significantly regulated pathways with known biological functions detected by DNB and DEGs.

Biological Function	DNB	DEGs (Cirrhosis vs. HGDNs)
Disease	Pathways in cancer (+) *	Pathways in cancer (+) *
	Viral carcinogenesis (+)	
	Hepatitis C (+)	
Immune response	Antigen processing and presentation (−) *	Antigen processing and presentation (−) *
	Natural killer cell-mediated cytotoxicity (−) *	Natural killer cell-mediated cytotoxicity (−) *
	RIG-I-like receptor signaling pathway (−)	T cell receptor signaling pathway (−)
	Cytosolic DNA-sensing pathway (−)	FcγR-mediated phagocytosis (−)
		FcεRI signaling pathway (−)
		Chemokine signaling pathway (−)
Cell adhesion	CAMs (−)	Focal adhesion (−)
		ECM-receptor interaction (−)
		Gap junction (−)
		Tight junction (−)
Cell motility		Regulation of actin cytoskeleton (−)
Cell growth and death	Cell cycle (+)	
Signal transduction	JAK–STAT signaling pathway (−) *	JAK–STAT signaling pathway (−) *
	TGF-β signaling pathway (+)	NF-κB signaling pathway (−)
		Notch signaling pathway (+)
		MAPK signaling pathway (−)
		Hedgehog signaling pathway (+)

* Represents the pathway which was in the both significantly regulated pathways detected by DNB and DEGs. (+) represents the pathway which was upregulated; (−) represents the pathway which was downregulated. CAMs: Cell adhesion molecules; ECM: Extracellular matrix; FcεRI: Fragment crystallizable ε receptor; FcγR: Fragment crystallizable γ receptor; JAK–STAT: Janus kinase–signal transducers and activators of transcription; MAPK: Mitogen-activated protein kinase; NF-κB: Nuclear factor-κB; RIG-I: Retinoic acid-inducible gene I; TGF-β: Transforming growth factor-β.

## Supplementary Materials

The following are available online at www.mdpi.com/2073-4425/8/10/268/s1, Figure S1: The criteria of DNB over all different stages of the HCC progression, Figure S2: The network of DNB, Figure S3: Kaplan–Meier overall survival curves for the HCV-induced HCC patients based on the expression of corresponding DNB members, Table S1: Detailed DNB members at LGDNs tipping point, Table S2: Functional enrichment of GO biological processes and KEGG pathways for the identified DNB, Table S3: Pathways enrichment analysis for DNB by IPA, Table S4: The degrees of DNB members in network.
