# Erratum: Lina Lu et al.; Low-Grade Dysplastic Nodules Revealed as the Tipping Point during Multistep Hepatocarcinogenesis by Dynamic Network Biomarkers. *Genes* 2017, *8*, 268

**DOI:** 10.3390/genes10050335

**Published:** 2019-05-02

**Authors:** Lina Lu, Zhonglin Jiang, Yulin Dai, Luonan Chen

**Affiliations:** 1 Key Laboratory of Systems Biology, CAS Center for Excellence in Molecular Cell Science, Shanghai Institute of Biochemistry and Cell Biology, Chinese Academy of Sciences, University of Chinese Academy of Sciences, Shanghai 200031, China; lulina@sibs.ac.cn (L.L.); jiangzhonglin@sibs.ac.cn (Z.J.); 2 Center for Precision Health, School of Biomedical Informatics, The University of Texas Health Science Center at Houston, 7000 Fannin St., Suite 820, Houston, TX 77030, USA; daiyulin@sibs.ac.cn; 3 School of Life Science and Technology, Shanghai Tech University, Shanghai 201210, China

The authors wish to make the following correction to their paper [1]. The first affiliation should be corrected to “Key Laboratory of Systems Biology, CAS Center for Excellence in Molecular Cell Science, Shanghai Institute of Biochemistry and Cell Biology, Chinese Academy of Sciences, University of Chinese Academy of Sciences, Shanghai 200031, China”.

The authors would like to apologize for any inconvenience caused. The change does not affect the scientific results. The manuscript will be updated, and the original will remain online on the article webpage.
