# Supplementary material for: Low-Grade Dysplastic Nodules Revealed as the Tipping Point during Multistep Hepatocarcinogenesis by Dynamic Network Biomarkers
Source: Genes (Basel). 2017 Oct 13;8(10):268. doi: 10.3390/genes8100268 (PMC5664118; doi:10.3390/genes8100268)
Supplement: Supplementary file 1 [file genes-08-00268-s001.zip › genes-218117_final_supplementary/genes-218117_final_supplementary.docx]

Supplementary Materials: Low-Grade Dysplastic Nodules Revealed as the Tipping Point during Multistep Hepatocarcinogenesis by Dynamic Network Biomarkers

Lina Lu ^1,†^, Zhonglin Jiang ^1,†^, Yulin Dai ^2^ and Luonan Chen ^1,3,^*

^1^ Key Laboratory of Systems Biology, CAS Center for Excellence in Molecular Cell Science, Innovation Center for Cell signaling Network, Institute of Biochemistry and Cell Biology, Shanghai Institutes for Biological Sciences, Chinese Academy of Sciences, Shanghai 200031, China; lulina@sibs.ac.cn (L.L.); jiangzhonglin@sibs.ac.cn (J.Z.)

^2^ Center for Precision Health, School of Biomedical Informatics, The University of Texas Health Science Center at Houston, 7000 Fannin St., Suite 820, Houston, TX, 77030, USA; daiyulin@sibs.ac.cn

^3^ School of Life Science and Technology, Shanghai Tech University, Shanghai 201210, China

***** Correspondence: lnchen@sibs.ac.cn

† These authors contributed equally to this work.


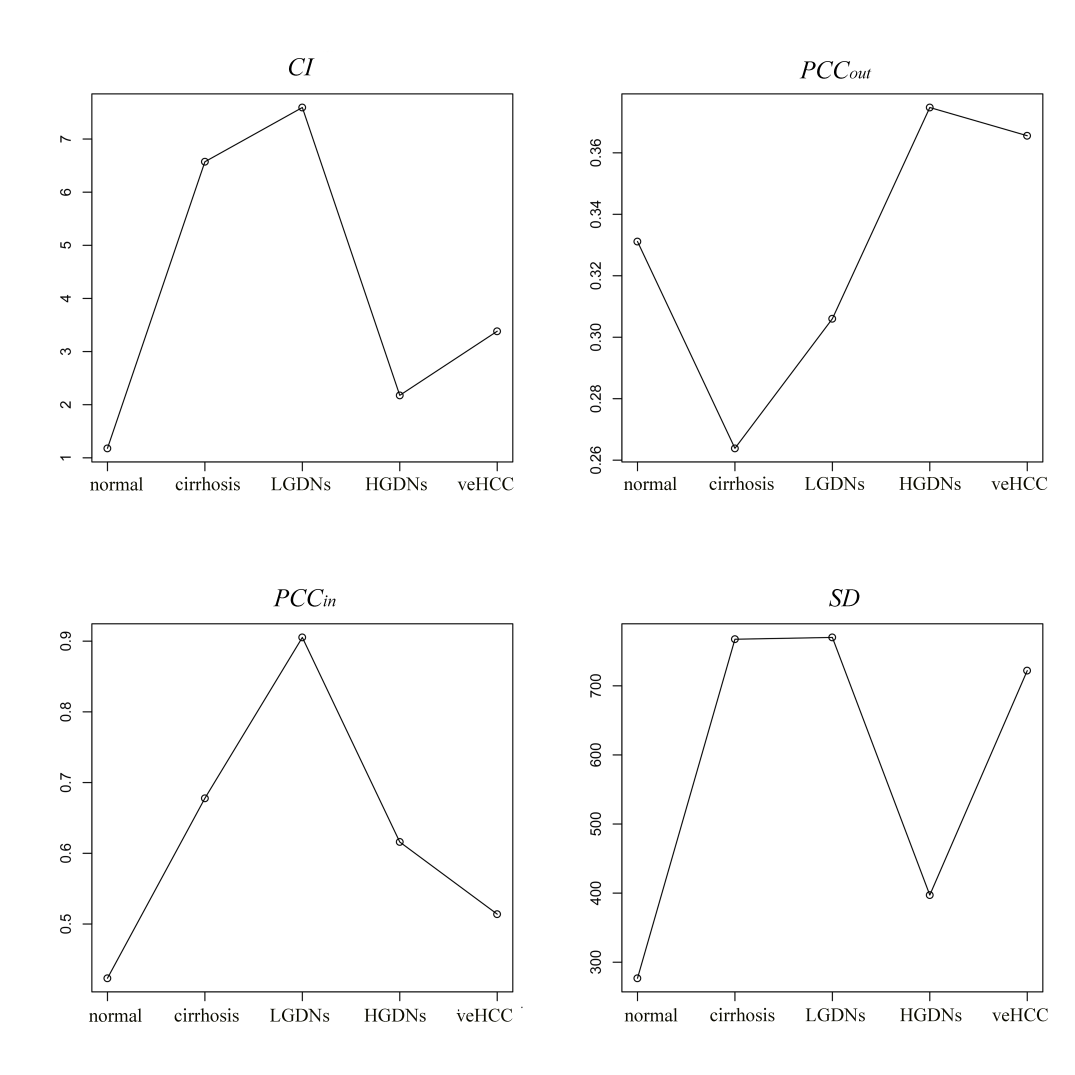


**Figure S1.** The criteria of DNB over all different stages of the HCC progression. (**A**) The composite index for the DNB (**B**) The average Pearson correlation coefficient between molecules inside and outside of DNB (*PCC_out_*). (**C**) The average Pearson correlation coefficient of all molecules pairs in DNB (*PCC_in_*). (D) The average standard deviation (SD) of DNB members. LGDNs: Low-grade dysplastic nodules; HGDNs: High-grade dysplastic nodules; veHCC: Very early HCC.

**
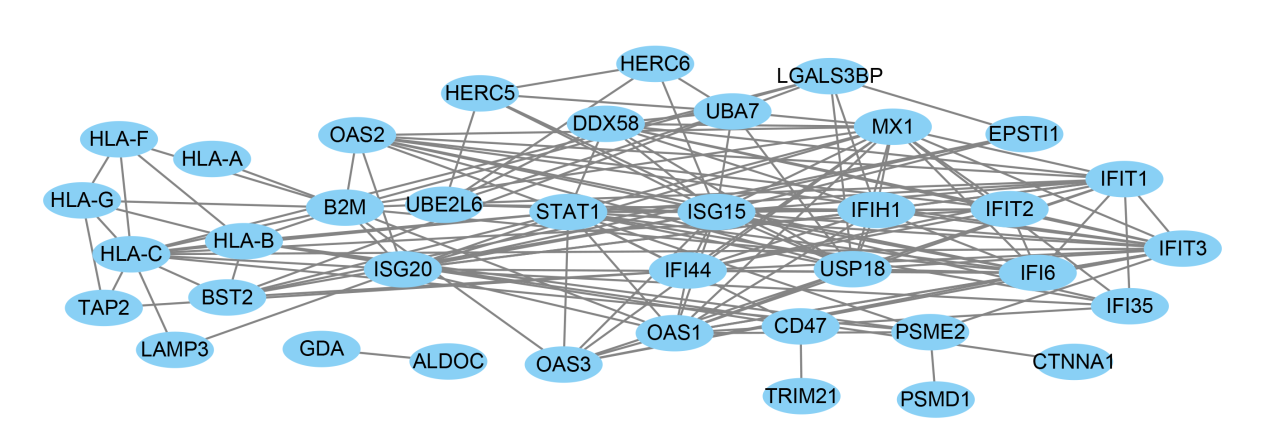
**

**Figure S2.** The network of DNB. Each node represents one DNB member. Edges correspond to protein–protein interactions based on STRING database.

**
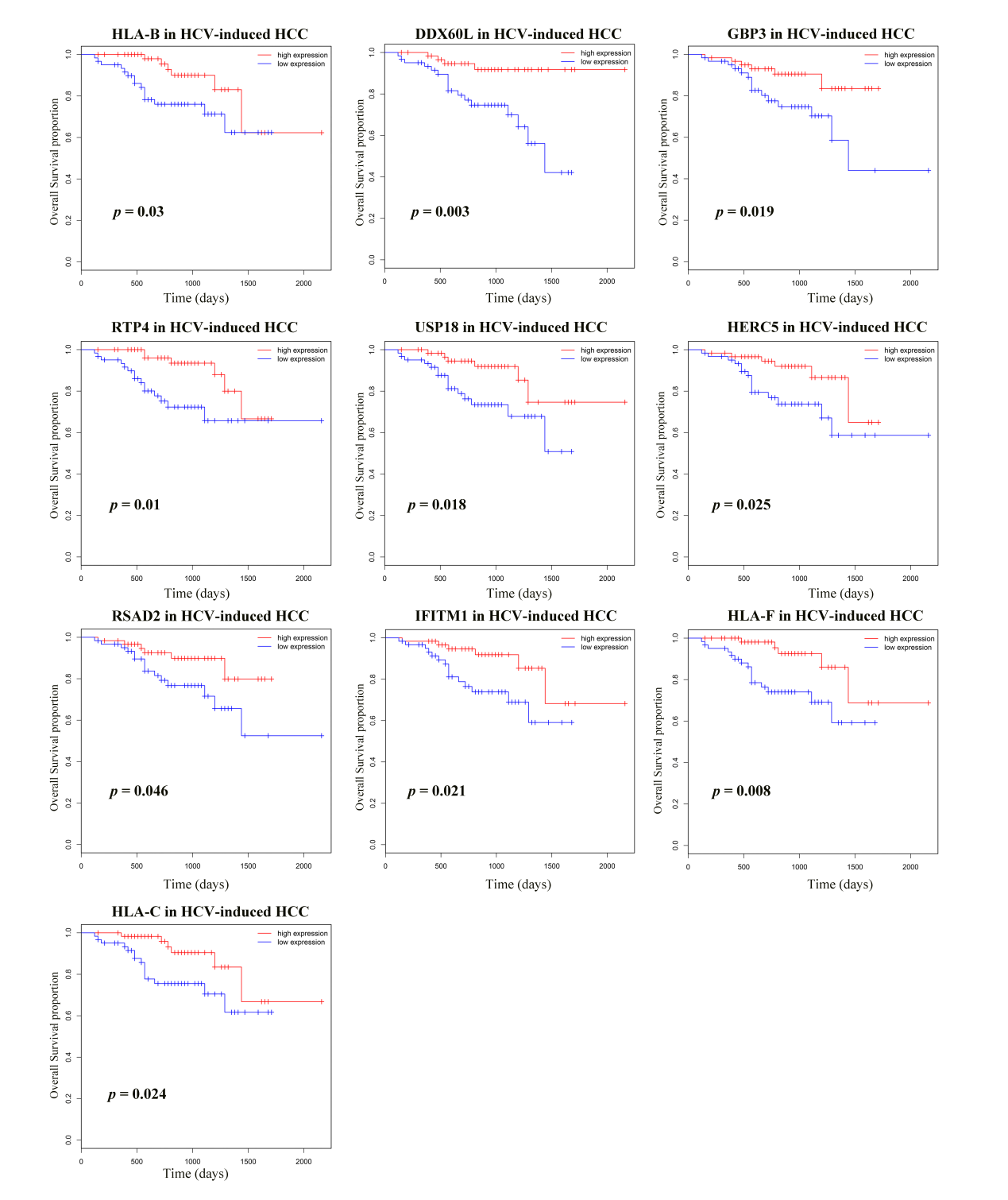
**

**Figure S3.** Kaplan–Meier overall survival curves for the HCV-induced HCC patients based on the expression of corresponding DNB members. Patients were divided into low- and high-expression group according to the median value.
